# Enhancing liver fibrosis measurement: Deep learning and uncertainty analysis across multi-center cohorts

**DOI:** 10.1016/j.jpi.2026.100653

**Published:** 2026-03-20

**Authors:** Marta Wojciechowska, Stefano Malacrino, Dylan Windell, Emma L. Culver, Jessica K. Dyson, on behalf of the UKAIH consortium, Jens Rittscher

**Affiliations:** a Nuffield Department of Medicine, University of Oxford, Oxford, UK; b Big Data Institute, University of Oxford, Oxford, UK; c Nuffield Department of Surgical Sciences, University of Oxford, Oxford, UK; d Perspectum Ltd., Oxford, UK; e Translational Gastroenterology and Liver Unit, John Radcliffe Hospital, Oxford, UK; f Translational and Clinical Research Institute, Newcastle University and Liver Unit, Freeman Hospital, Newcastle upon Tyne, UK; g Department of Engineering Science, University of Oxford, Oxford, UK

**Keywords:** Digital pathology, Liver, Collagen staining, Collagen quantification, Quality assurance, Uncertainty

## Abstract

Digital pathology enables large multi-center studies of histological specimens, but differences in staining protocols and slide quality can compromise the comparability of quantitative results. We analyzed 686 PicroSirius Red-stained liver biopsies from 4 independent cohorts spanning more than 20 clinical sites to assess how stain variability affects automated fibrosis quantification and model uncertainty. An U-Net ensemble was trained to segment collagen and to estimate pixel- and tile-level predictive uncertainty. Across markedly heterogeneous staining conditions, the ensemble achieved strong segmentation performance (Dice 0.83–0.90) and produced informative uncertainty maps that identified artifacts and out-of-distribution regions. Epistemic uncertainty values were typically below 0.002, providing a practical criterion for flagging unreliable predictions. Our results demonstrate that ensemble-based uncertainty estimation complements stain-standardization efforts by quantifying prediction confidence directly from model outputs, improving the reliability and interpretability of collagen proportionate-area measurements across multi-center datasets. This framework supports more trustworthy and reproducible digital-pathology workflows for fibrosis assessment and other histological applications.

## Highlights

• A retrospective cohort of liver biopsies collected from over 20 healthcare centers has been assembled.
• The cohort is characterized on the basis of collagen staining used for liver fibrosis assessment.
• We developed and applied a computational pipeline to quantify collagen in liver histology across the cohorts.
• Uncertainty estimation is evaluated as a method to build trust in deep-learning based collagen predictions.

## Graphical abstract

**Figure:**
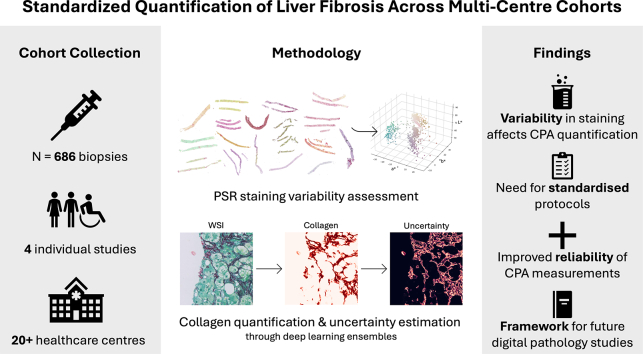

## Introduction

Digital pathology enables scalable, multi-center studies of histological biomarkers, yet systematic differences in slide preparation and image acquisition continue to hinder reproducible quantification. In this work, we examine how staining heterogeneity affects automated collagen quantification and explore how ensemble-based predictive uncertainty can be used to interpret and manage unreliable measurements. Using PicroSirius Red (PSR)-stained liver biopsies collected from 4 independent cohorts across more than 20 clinical sites, we develop a deep-learning framework that combines stain characterization with model-ensemble uncertainty estimation to improve the reliability and interpretability of collagen proportionate-area (CPA) measurements in liver fibrosis.

Fibrosis is increasingly recognized as a core functional component of the tissue microenvironment, and CPA is an established image-based metric derived from whole-slide images (WSIs) used in liver research.^1^ CPA has been proposed as a trial endpoint in liver disease and is associated with long-term outcomes in metabolic-associated fatty liver disease and alcoholic liver disease.^2^, ^3^, ^4^ However, CPA reflects only collagen burden rather than architectural patterns of fibrosis, and its clinical utility depends critically on consistent detection and quantification. Despite its promise, the application of CPA as an imaging biomarker faces substantial challenges. Published findings often lack consistency, largely due to variations in collagen detection and quantification methodologies,^5^ which undermine comparability across centers.

The literature on stain standardization is extensive. Early approaches relied on stain deconvolution and stain-vector estimation,^6^, ^7^, ^8^ but assumptions such as white-light illumination limit their accuracy for WSI.^9^ Later methods introduced histogram-matching and color-transfer techniques^10^^,^^11^ and, more recently, generative frameworks such as CycleGAN and related autoencoders.^12^, ^13^, ^14^, ^15^, ^16^ These models can produce visually convincing stain transformations, but their biological fidelity and impact on quantitative analysis remain uncertain.

Automated quality-control tools such as HistoQC are highly effective for detecting slide-level artifacts,^17^ but they do not quantify intercohort staining variability or model confidence. To address these gaps, we combine color-space analysis with ensemble-based uncertainty estimation, enabling both cohort-level assessment of staining heterogeneity and model-level assessment of prediction reliability.

This article first presents cohort-level color analyses (Section Color characterization of digital slides) that document staining heterogeneity, then describes the U-Net ensemble and uncertainty estimation approach (Section Computational methods), and finally evaluates how uncertainty maps relate to segmentation performance and practical quality control (Section Results).

The following sections provide a detailed examination of the sources of measurement variability that motivate our approach.

### Causes of uncertainty in CPA measurement

First, it must be acknowledged that any biopsy sample may not be representative of the examined organ as a whole. It is commonly estimated that a liver biopsy captures approximately 1:50,000 of the organ's volume.^18^ Given the known heterogeneity of fibrosis distribution in chronic liver disease, a single-slice CPA measurement may under- or overestimate total collagen content. This limitation affects all histology-derived metrics and predictions and has been addressed elsewhere.^19^ Whereas we focus here on quantifying uncertainty within digitized slide images, a full evaluation of sampling variability in CPA lies beyond the scope of this study. However, from the moment of biopsy acquisition, there are still a number of confounding factors that need to be considered to obtain a reliable measure of the collagen area in the analyzed sample.

The process of CPA measurement can be broken down into three separate detection and quantification tasks: (1) collagen in-situ localization (i.e., chemical staining), (2) the acquisition of the digital image, and (3) the quantification of the stained area relative to the area of the tissue section. Each of these processes is subject to its own sources of error, which collectively contribute to the overall uncertainty of the measurement.

### In-situ localization of collagen

Liver fibrosis is a condition characterized by the pathological deposition of collagen fibers within the liver parenchyma, primarily in portal and perisinusoidal areas, and is therefore assessed using collagen-binding stains. Other connective tissue fibers, such as elastin fibers are also present in fibrotic structures. In comparison, their quantity is typically small.^20^ Several staining for identifying connective tissue in general and collagen specifically have been independently developed and traditionally used in pathology studies and in the clinic.^5^ These include: PSR, Masson's Trichrome (MT) staining for connective tissue, van Gieson's stain, orcein, and others. Each of these staining is used in pathology to make the structure of connective fibers visible and can be used to visually assess the grade of fibrosis. However, the only histochemical stain which is known to be collagen-specific and so can be used to quantify collagen is Sirius Red.

PSR is a staining developed in the 1960s with the purpose of selectively staining collagen in histology samples.^21^ The primary agents in this staining are Sirius Red F3B (CI 35780, Direct red 80) and picric acid (2,4,6-trinitrophenol, TNP). In the presence of picric acid, Sirius Red F3B selectively binds with type I collagen (thicker collagen fibers), and type III collagen (thinner fibrillar collagen/reticulin fibers).^22^ Because of the selective staining for type I and III collagen fibers and its sensitivity to fine collagen strands, the PSR protocol is preferred for automatic quantification of collagen in histology slides. Another commonly used protocol for clinical assessment of liver fibrosis is MT, in which collagen and other connective fibers are stained blue. In this article, only Sirius Red-stained slides were available for the development of digital pathology methods.

**Fig. 1:**
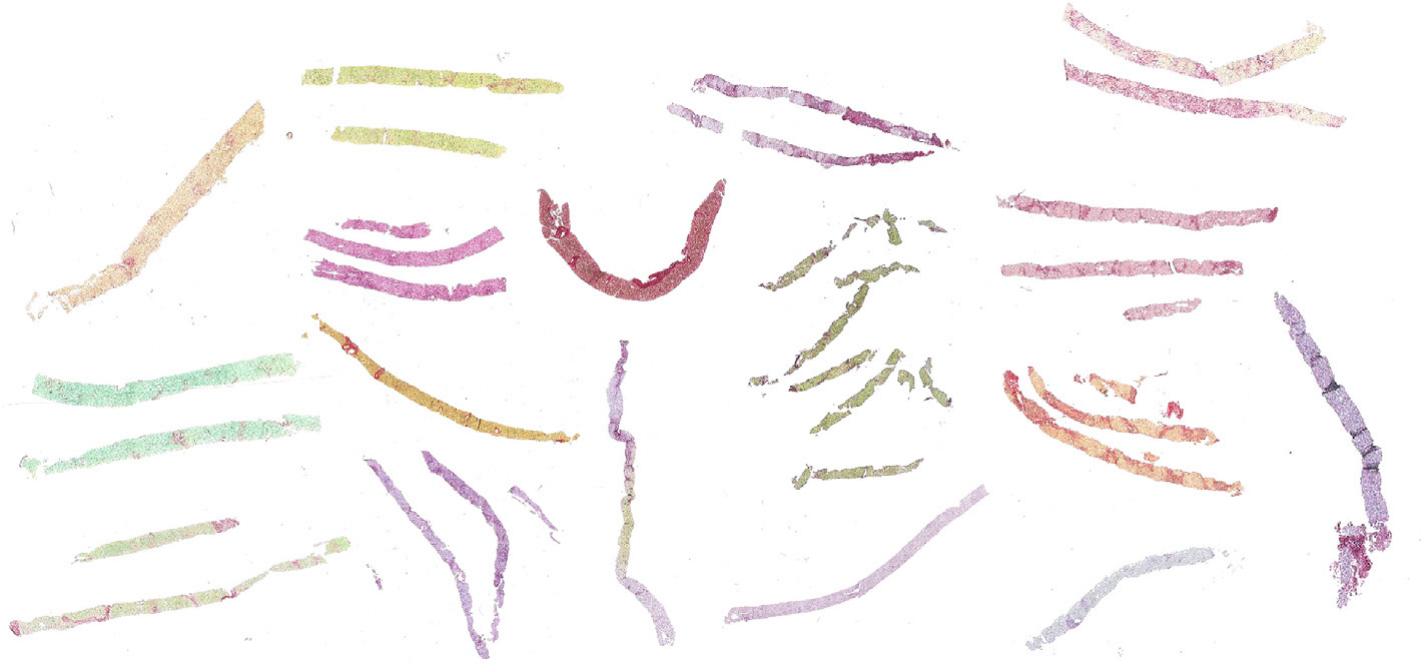
A visual representation of a selection of Sirius Red-stained liver slides from the UK-AIH subcohort. This image illustrates how the use of different protocols for PSR collagen staining results in dramatic differences in slide appearance within the cohort collected retrospectively from 20 NHS hospitals. The images are not to scale. In each digital slide, the background was removed and colors adjusted through histogram equalization for visual clarity.

In spite of its wide-spread use as a collagen-specific stain, there is not a single standard protocol for PSR staining, which is used globally across institutions. The classical form of PSR staining includes only the mixture of Sirius Red and picric acid for collagen detection.^21^ However, labs often add additional stains to the same tissue slice, to simultaneously examine other forms of tissue pathology. Commonly, two other stains may be added: a nuclear stain (a hematoxylin) and a cellular plasma stain (typically a green dye). This lack of PSR staining standardization between healthcare institutions leads to very significant differences in the color of the stained slides. This phenomenon can be well observed within the UK-AIH cohort, where 20 NHS UK hospitals contributed liver FFPE slides prepared within their pathology labs (Fig. 1). The figure illustrates how deep the visual differences between PSR-stained slides can be when comparing samples collected at different sites.

Whereas PSR staining is known to create differential contrast between collagen strands and other tissue components, the exact sensitivity and specificity of PSR stain binding to collagen in a liver tissue section is not known.^22^ Additionally, parameters such as the freshness of the specimen, stain concentration, stain exposure time, and ambient temperature can all affect the intensity of staining. It is not clear how the interactions of different chemicals and even the order in which different stains are applied affects the sensitivity and specificity of stain–collagen binding. Finally, histological stains are known to fade over the course of years and therefore, retrospectively collected slides may not be comparable to freshly obtained ones in terms of color intensity.

### Digital sampling

The thickness of an individual collagen fiber is well below the *x,y* imaging resolution of a typical WSI scanner.^23^^,^^24^ Collagen fibers which can be seen in WSIs are aggregates of many collagen fibrils and are characterized by highly complex fractal-like shapes. Because of this fractal-like appearance of the analyzed shape, the measure of collagen area is intrinsically tied to the *x,y* resolution of the microscope objective lens and the size of the pixel of the imaging sensor.^25^ The dependency of the quantified collagen area on the imaging magnification is significant, which is why fractal metrics of dimensionality have been successfully applied to predict the overall stage of fibrosis.^26^

Beyond the imaging resolution in the xy-plane, parameters such as slice thickness, lighting conditions, and sensor exposure time directly affect the intensity of the color of the digitized slide.^27^ Clinical WSIs are typically acquired at either 20× or 40× magnification, which corresponds to *x,y* pixel sizes of approximately 0.5 × 0.5 μm, and 0.2 × 0.2 μm, respectively (see Table 2). In contrast, typical clinical biopsies are sliced at the thickness range between 4 and 10 μm. Hence, a WSI pixel can contain a wide range of collagen thicknesses within the corresponding *z* volume of the tissue.^28^

The final color intensity of an imaged pixel is therefore a function of:(1) the thickness of the tissue slice, (2) the thickness of the imaged collagen fiber in the tissue volume, and (3) the relative intensity of collagen staining.

### Segmentation errors

**Fig. 2:**
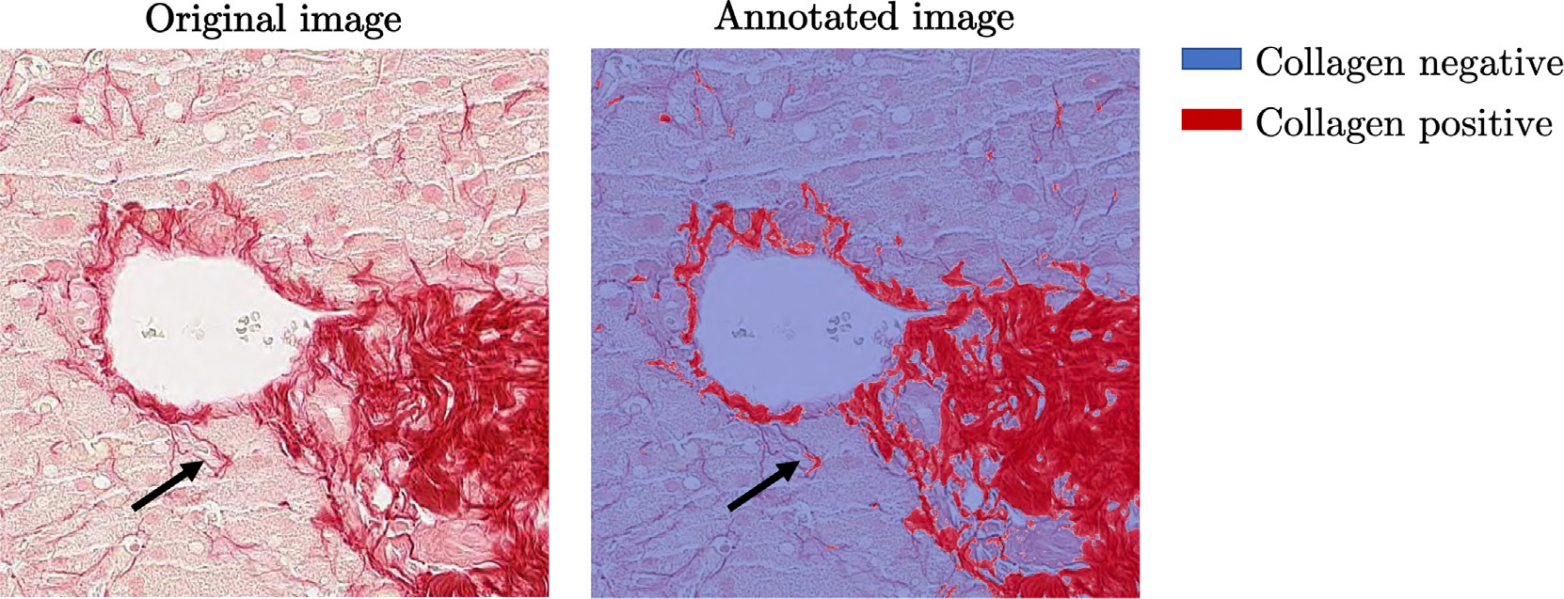
Limitations of creating human annotations of collagen fibers in microscopy images. The task is highly subjective and characterized by high inter- and intraobserver variability, particularly for thin collagen strands and in tissue slides with poor staining quality. Whereas regions with thick, intensely stained collagen are captured, thinner fibers were missed by the annotator (MW).

Finally, at any resolution, the results of CPA quantification depend on the quality of the segmentation of collagen fibers. Fig. 2 illustrates the difficulty of annotating collagen on an example tile from a PSR-stained liver biopsy specimen. For now, we focus on the data of the PREV study and only consider Sirius Red staining. The stain is present in the entire tissue section and can show some spatial heterogeneity. Typically, collagen fibers have an intensely red color. But, it can be seen that other regions within the tissue parenchyma (cell cytoplasm and even more so cell nuclei) are also colored pink. Unspecific staining is a factor that needs to be taken into account. Compression artifacts are another source of noise that needs to be considered. This illustrates why annotating collagen fibers in liver histology slides is burdened with high inter- and intraobserver variability.^29^

Performing manual or even semi-automatic annotations of collagen area is a tedious task, which can easily be automated by the employment of image analysis methods. Simple annotation tools often rely on the manual selection of a pixel intensity threshold by the annotating pathologist; such pipelines are known to be vulnerable to stain intensity gradients, which are often found in histology slides. Deep learning segmentation models can be trained against pathologist segmentations to be robust to minor and even major differences in stain color.

## Materials and methods

### Cohorts and data acquisition

Four separate datasets of liver digital slides are used in this article, henceforth, referred to by the names of their respective original studies. Table 1 contains a brief summary of the cohort characteristics.

**Table 1:** An overview of the study cohorts.

Cohort	*N* slides	*N* sites	Disease	Country
CALM^30^	153	2	Mix	UK
HepaT1ca^31^	44	1	Liver Cancer, MAFLD	UK
PREV^32^	279	1	MAFLD	USA
UK-AIH^33^	210	20	AIH	UK

In total, 686 digitized biopsies of mixed etiologies were gathered and analyzed from a combined total of over 20 clinical sites. In each of the studies, liver biopsies or surgical resections were collected as part of standard care, with the exception of the PREV cohort, which consists entirely of volunteers. Slides stained with H&E and PSR staining were included in the analysis.

The samples within each of the three prospective cohorts in this article: CALM, HepaT1ca, and PREV have been processed according to internally agreed processing and staining protocols. The specimens from all these studies have been stained with automatic stainers. In contrast to the above cohorts, the UK-AIH study is a retrospective study and slides dating back to 1998 from 20 hospital sites in the UK were selected. The FFPE slides in this cohort were processed according to internal standards within each of the participating hospitals at the time they were collected.

The CALM (ISCRTN39463479), HepaT1ca (NCT03213314), and PREV (NCT03142867) cohorts were made available by Perspectum Ltd., Oxford, UK. The UK-AIH cohort (IRAS ID: 144806) was made available by the UK-AIH consortium funded by the NIHR Rare Diseases Translational Research Collaboration (RD-TRC) and coordinated by Newcastle University and Newcastle upon Tyne Hospitals NHS Foundation Trust. The UK-AIH study cohort was supported by LiverNorth, a UK-based patient support group and registered charity (No. 1087226). Further information about study design and data acquisition is available in the respective original publications.^30^, ^31^, ^32^, ^33^

### Slide digitization

The glass slides were digitized using WSI scanners. Each cohort was scanned internally using parameters chosen by the individuals conducting the respective studies. Key parameters of the image acquisition are summarized in Table 2.

**Table 2:** Overview of digital slide parameters for each of the cohorts.

Cohort	Scanner	Pixel size: 20× magnification [μm]	File format	Compression
CALM	Hamamatsu C12000–02	0.4533, 0.4533	*.ndpi	JPEG *level unknown*
HepaT1ca	Hamamatsu C12000–02	0.4533, 0.4533	*.ndpi	JPEG *level unknown*
PREV	Aperio *model unknown*	0.4954, 0.4954	*.svs	Aperio JP2000 RGB
UK-AIH	Leica SCN400	0.5000, 0.5000	*.scn	JPEG *level unknown*

Scanners from several WSI manufacturers were used for data acquisition. WSI systems from different vendors are equipped with different optical instruments and digital image sensors. These differences in hardware mean that the WSIs were acquired at slightly different resolutions, given the same nominal magnification (e.g., 20×). Similarly, scanning the same histology slide with two WSI scanners from different vendors produces digital images with slightly different colors.^8^ This can be a result of both hardware differences and the color calibration of a particular scanner. Each vendor also typically uses a proprietary image format.

All slides were processed using the vendor-neutral libraries: OpenSlide^34^ and wsi-reader.^h^ Wsi-reader is a Python library developed by SM within the Rittscher group at the University of Oxford, designed to offer a unified interface for the most common WSI libraries, such as openslide, tifffile,^i^ and Philips Pathology SDK.

### Sirius Red staining for collagen

To assess the stains used in each of the respective studies in the absence of staining protocols, stain deconvolution was performed on selected digitized slides from each cohort.^6^ Stain deconvolution is a transformation of the image from the RGB colorspace into a colorspace in which each of the three image channels encodes the proportion of a color of a single stain. This processing was performed using QuPath software.^35^ Whereas we cannot definitely identify particular stains or reconstruct staining protocols retrospectively using stain deconvolution, we are here using it to detect and document atypical color profiles.

**Fig. 3:**
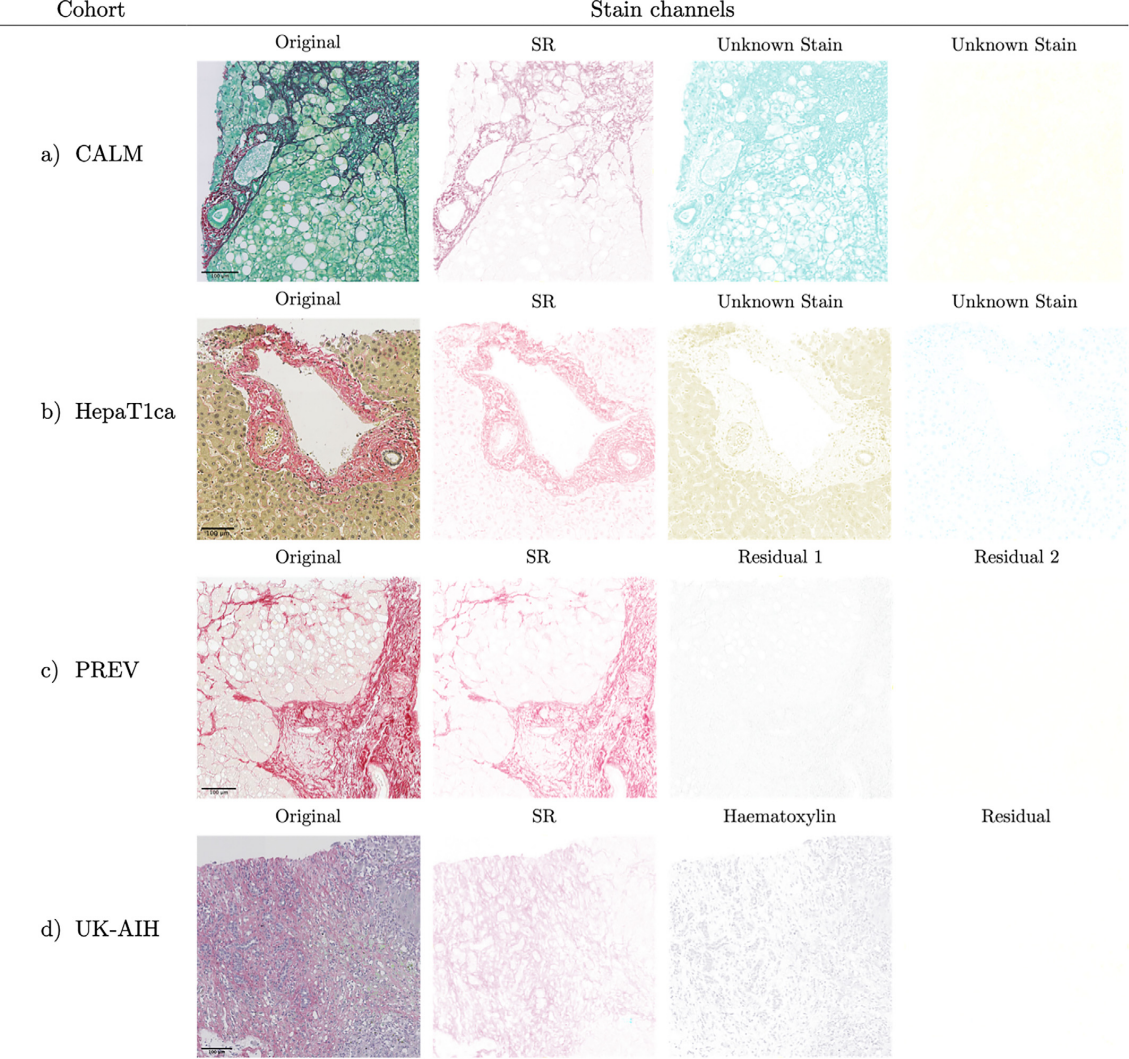
The results of stain deconvolution on example digital PSR slides from the analyzed cohorts. (a) CALM: The black color of some collagen fibers is a result of non-specific binding of an unknown green stain to fibers stained with Sirius Red. The slides contain a pale-yellow residuum. (b) HepaT1ca: Sirius Red and two unknown stains, olive and blue. Upon close inspection, one can see that cell nuclei are non-specifically stained red along with the collagen. (c) PREV: Only Sirius Red stain was detected. (d) UK-AIH: Slide stained with Sirius Red and a purple hematoxylin with an empty residual channel. (For interpretation of the references to color in this figure legend, the reader is referred to the web version of this article.)

**Fig. 4:**
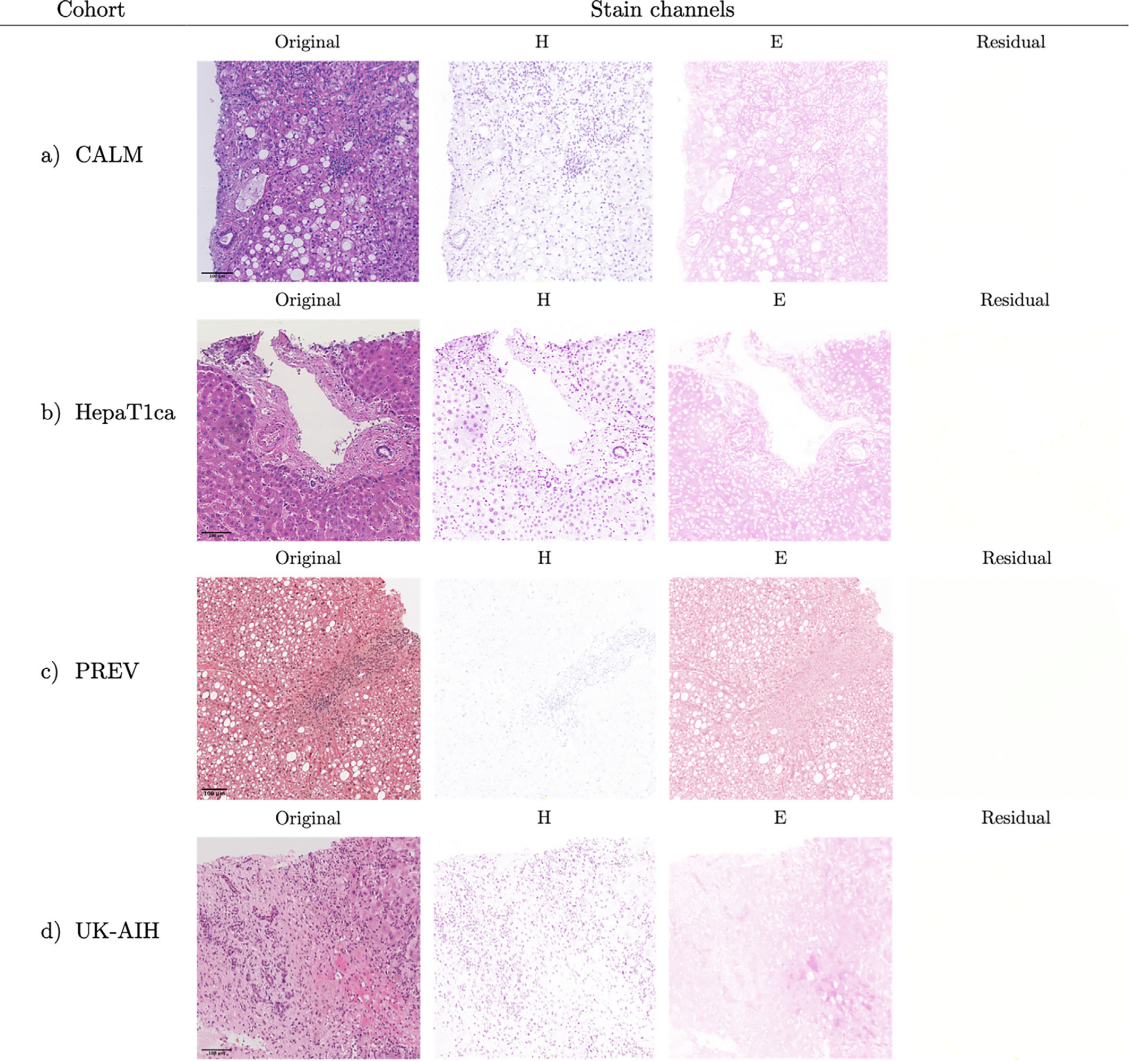
The results of stain deconvolution on example digital H&E slides from the four study cohorts. In each of the cohorts, a hematoxylin and an eosin were used to produce H&E slides. The residual channel is empty or nearly empty in all the slides. Slight differences in stain hue of both hematoxylin and eosin, as well as scan background color can be seen between samples from different cohorts.

The stain deconvolution of the Sirius Red staining, shown in Fig. 3, is used as an example to demonstrate the staining variability. Although a red collagen dye was used in each of the centers, it is obvious that different counterstains were used. This is in stark contrast to the results of stain deconvolution applied to the corresponding H&E slices from the same samples (Fig. 4).

H&E staining is more standardized across study cohorts than PSR. In each cohort only hematoxylin and eosin are used with no additional counterstains. In PSR-labeled slides, the number of dyes used was ranging from one (just Sirius Red) to three (i.e., Sirius Red and two additional stains). In the case of the non-homogeneous UK-AIH cohort, different numbers and colors of stains were used for PSR staining.

### Color characterization of digital slides

**Fig. 5:**
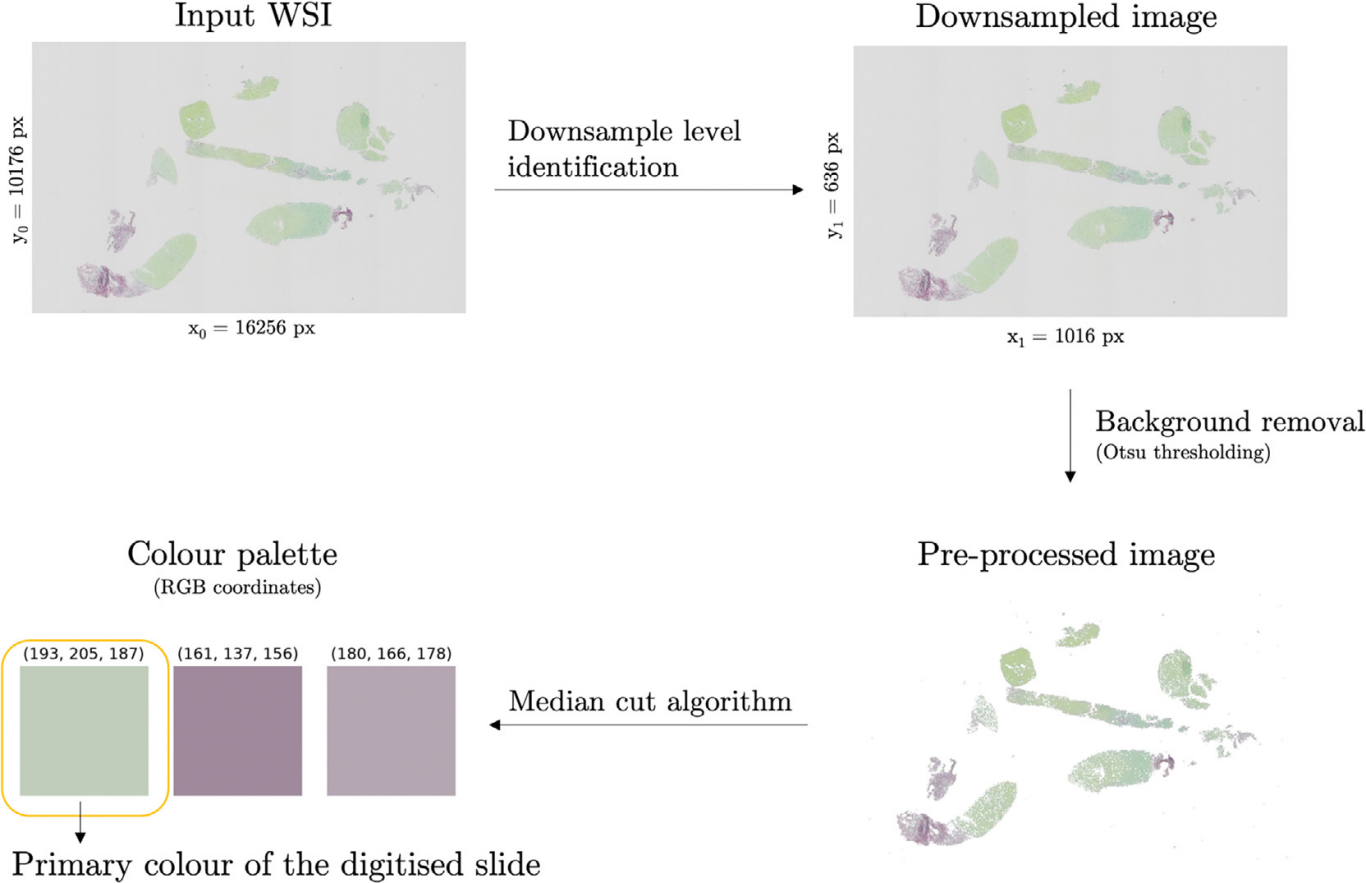
Identification of the primary color of a digital slide. First, an appropriate downsampled level is identified in the WSI pyramid. The slide image is then read from the downsampled level of the WSI file. Tissue/background intensity-based threshold is identified with the Otsu thresholding algorithm (applied to the grayscale-converted image). Background pixels are replaced with white values. Finally, the median cut algorithm is applied to the preprocessed image to identify the primary RGB value in the digital slide.

In order to visualize the spectrum of Sirius Red-stained digitized FFPE section appearances, we here propose the concept of a primary color of a digital slide (Fig. 5).

One of the most commonly used approaches to quantization of colors in digital images is through the employment of median cut algorithm.^36^ Median cut algorithm is a method of sorting data of an arbitrary number of dimensions into a series of sets by recursively cutting each set of data at the median point along the longest dimension. When applied to an image histogram, it outputs the most commonly occurring pixel values. Here, the WSI is first preprocessed by the extraction of a downsampled version of the image and background removal. A pixel intensity threshold between tissue and background is established using the Otsu method applied to a gray-level version of the image.^37^ All pixels of value above the established intensity threshold are replaced with white values. Median cut algorithm is applied to the preprocessed image to extract a three-color color palette, and the RGB values of the most commonly occurring color encode the primary color of the digital slide.

**Fig. 6:**
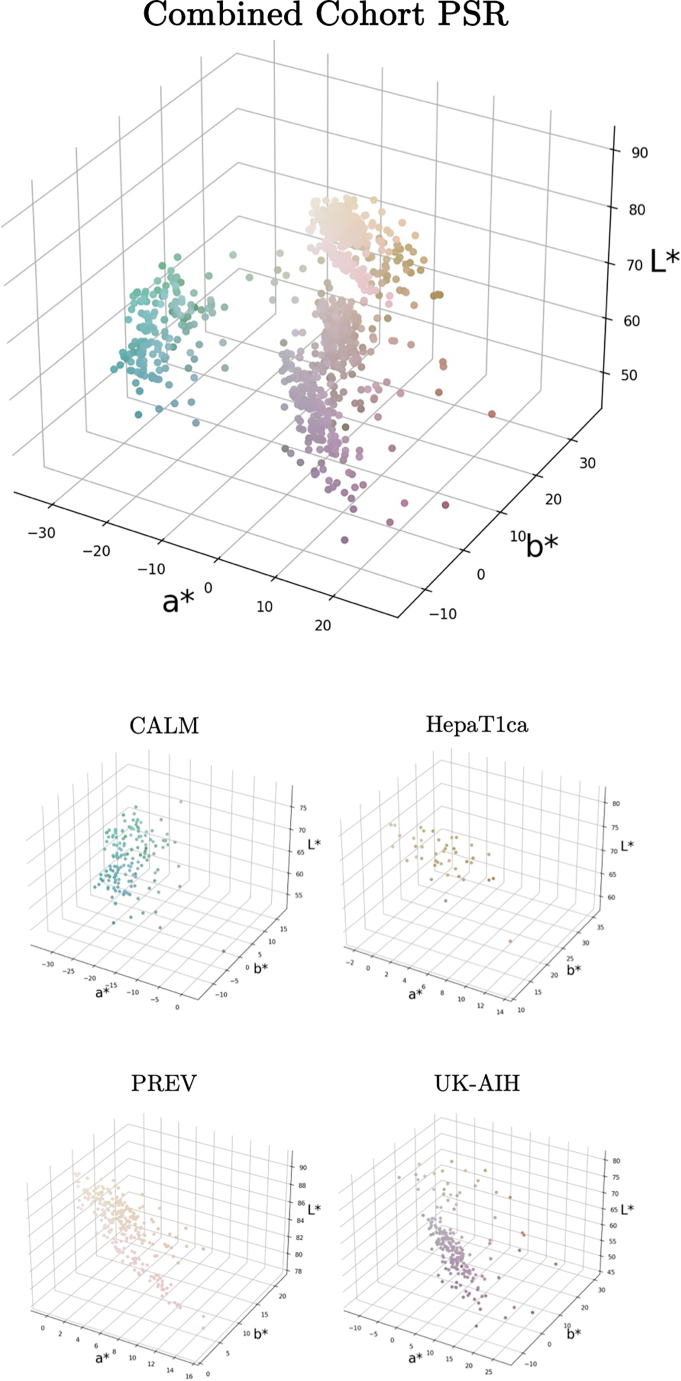
Primary color distribution in digital slides of PSR-labeled sections. Samples from the individual cohorts form separate color clusters.

**Fig. 7:**
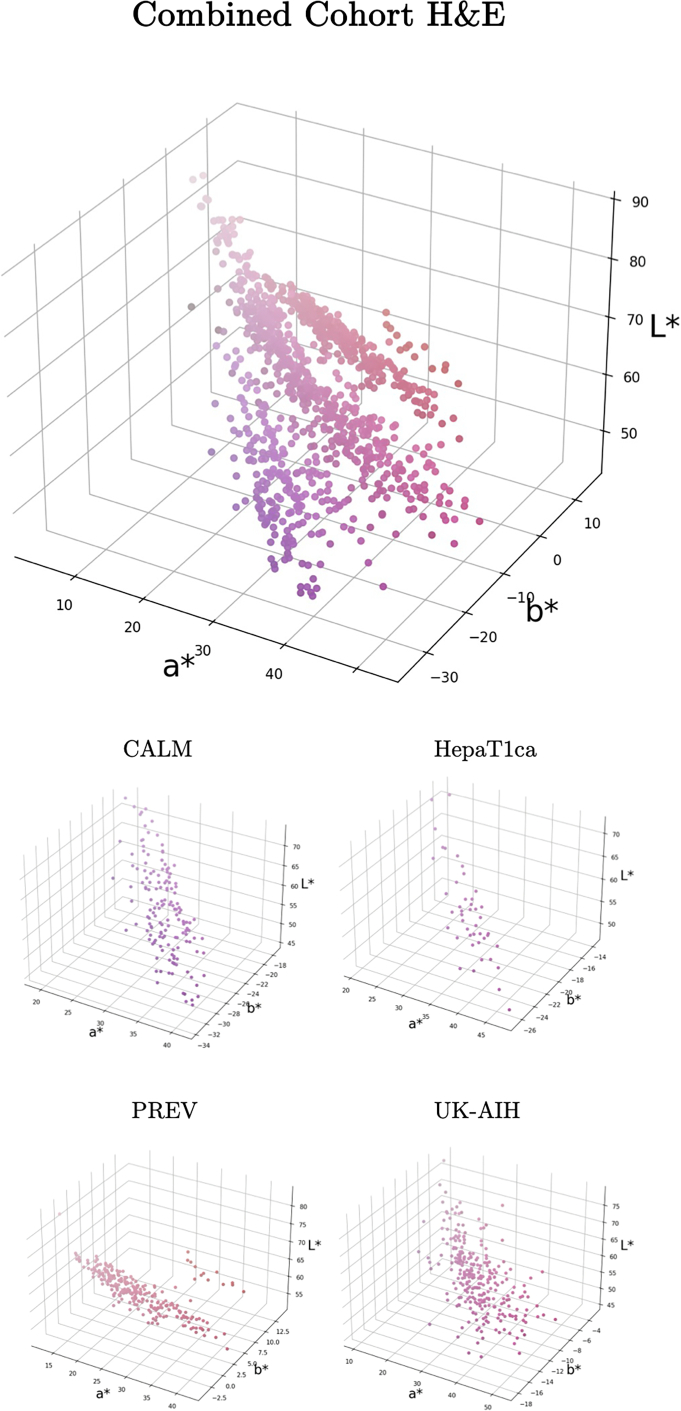
Primary color distribution in digital slides of H&E-stained sections.

The primary color has been computed for all the SR and H&E-stained digitized slides available to this project. For purposes of comparison, the RGB values of the primary slide colors were converted to the more perceptually uniform CIELAB colorspace.^38^ The CIELAB colorspace expresses each color as three values: L^⁎^ for lightness, a^⁎^, where negative values indicate green and positive values indicate red, and b^⁎^, where negative values indicate blue and positive values indicate yellow. The results of primary color estimation for the WSIs from all four cohorts are shown in Fig. 6, Fig. 7. These distributions quantitatively demonstrate the broad variability in Sirius Red staining across centers, complementing the qualitative examples in Fig. 1.

## Computational methods

### Experimental data

**Fig. 8:**
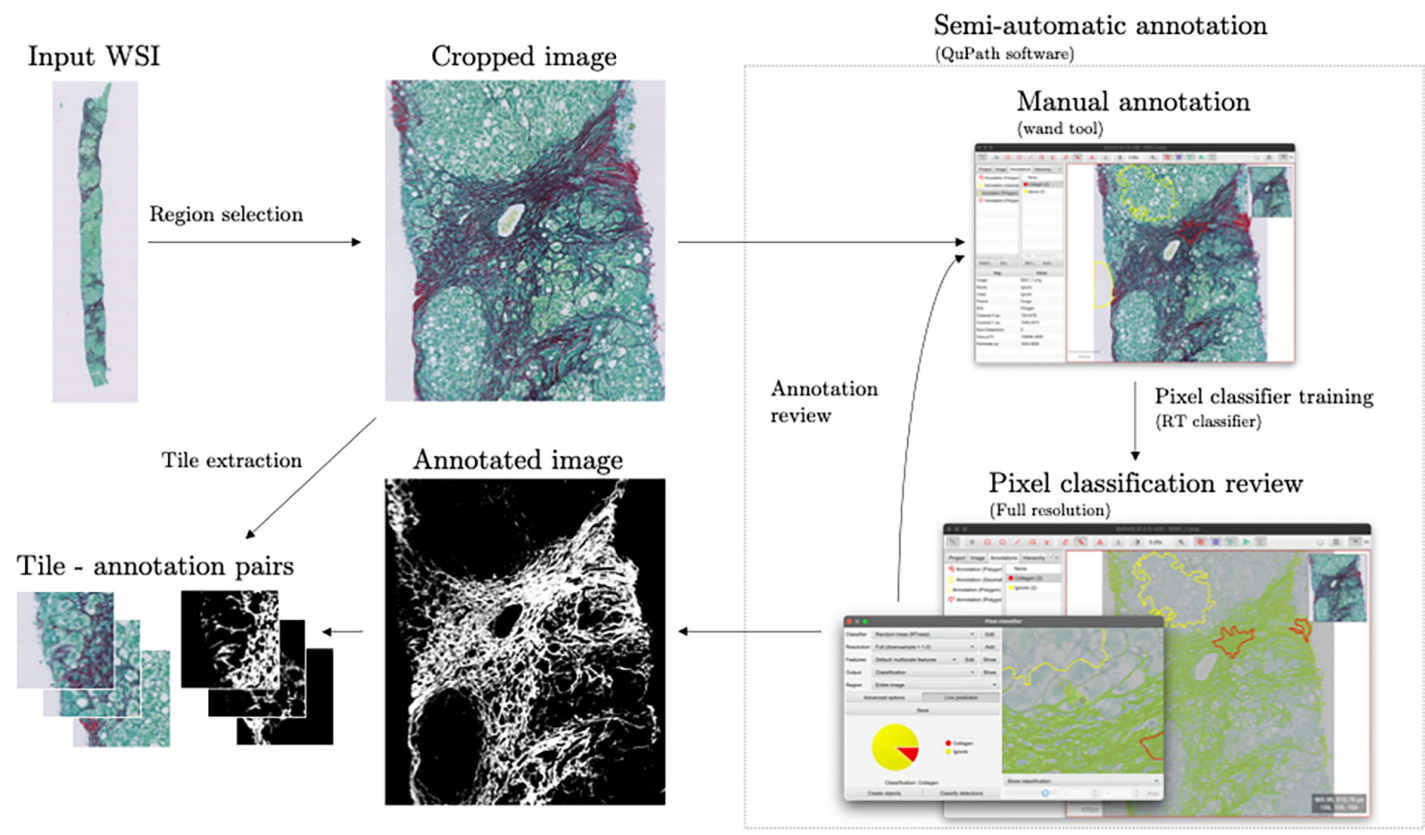
Collagen annotation with human in the loop. Firstly, a representative region is cropped from the full resolution level of the WSI file. The annotator trains a built-in random trees (RTs) pixel classifier to categorize individual pixels within the image as either: “collagen” or “ignore”. The results of the RT classification are carefully examined visually. If needed, the human annotations are refined, and the process is repeated until the classifier produces a satisfactory pixel-level classification for the entire image. The annotation is then exported as a binary mask.

Table 3 summarizes information about the training and validation data in this article. Cases from the three training cohorts (CALM, PREV, and UK-AIH) were manually selected using the obtained PSR stain distribution (Fig. 6) as a guide, to maximize the variation in color in the experimental dataset. In total, 38 WSIs of liver biopsy sections were densely annotated by MW using QuPath software.^35^ The pipeline for semi-automatic annotation of the slides with human in the loop is shown in Fig. 8. Tiles containing less than 5% of annotated collagen area were discarded from the training and validation set to improve the stability of model training. All tiles from the HepaT1ca study were retained as an independent (unseen) validation set. A total of 2367 annotated 512 × 512 px tiles were used in the analysis. Of these, 1970 were used for model training and 397 for model validation.

**Table 3:** A summary of training and validation data for each of the study cohorts. Images from CALM, PREV, and UK-AIH cohorts were used for method development. HepaT1ca was retained as an independent validation dataset.

Cohort	CALM	PREV	UK-AIH	HepaT1ca
*N* annotated cases	12	13	13	5
*N* train tiles	442	1172	356	–
*N* validation tiles	53	141	52	151
Mean collagen %	21%	15%	24%	–

### Segmentation models

The U-Net is a popular deep learning model used for medical image segmentation.^39^ We trained three variants of the U-Net model for the collagen segmentation task using the implementation available at.^j^ The first two models (here called U-Net Tiny and U-Net Mini) are each downsized versions of the original U-Net. An Attention U-Net^40^ was trained for the same task. The parameters of the individual networks are summarized in Table 4.

**Table 4:** A summary of the parameters of the trained segmentation models.

Parameter	U-Net Tiny	U-Net Mini	Att U-Net
Depth	3	3	3
Number of filters	[8, 16, 32]	[32, 64, 128]	[32, 64, 128]
Stacked layers (Down)	2	2	2
Stacked layers (Up)	2	2	2
Trainable parameters	32,665	517,729	612,691

Each of the trained segmentation networks has an input, which matches the size of 512 × 512 px RGB image tiles. The ReLU activation function was used in each segmentation model. U-Net Tiny and U-Net Mini used max pooling as the pooling strategy and a sigmoid activation function at the network output. Each of the models used bilinear unpooling. The models were trained using binary cross-entropy loss and Adam optimizer. Each of the networks was trained from scratch with a randomized initialization seed and a batch size of 8. A Quadro RTX 6000 GPU core was used for all the experiments conducted.

### Uncertainty estimation

In order to estimate the real uncertainty of PSR measurement, one would need to perform an uncertainty estimation for each of the steps in sample processing and image acquisition separately. Unfortunately, as only digitized slides were made available to this project, such an analysis goes beyond the scope of this article. Here, we propose a way of estimating the uncertainty inherent to the digital image itself, which can be inferred by the image segmentation deep network.

Uncertainty estimation in the context of deep learning can improve the quality of predictions.^41^^,^^42^ Bayesian inference is a statistical framework, which can be used to quantify the uncertainty present in deep-learning models. The concept is based on Bayes' theorem, which links the posterior distribution of our observations to the prior probability of the hypothesis and the likelihood of the data given the hypothesis:

$$P\left(A|B\right)=\frac{P\left(B|A\right)P\left(A\right)}{P\left(B\right)}$$

where: P(A|B) is the posterior probability of hypothesis A given the observed data B, P(B|A) is the likelihood of the observed data B given the hypothesis A, P(A) is the prior probability of hypothesis A, and P(B) is the marginal likelihood of the observed data B.

The Bayesian framework can be used to quantify uncertainty. However, exact Bayesian inference with deep neural networks is computationally intractable. It can be approximated by Bayesian neural networks (BNNs), which are neural networks with probabilistic weights and biases. In the BNN framework, the weights and biases of a network are treated as random variables with a prior distribution over their values. Bayesian inference is then used in the process of training the network to update the prior distribution with new data. The posterior distribution over weights and biases obtained during network training can be used to compute the predictive distribution of the model. This predictive distribution of the model is a representation of the range of possible model outputs given an input. The uncertainty of the model can be inferred from the model's predictive distribution and can be a measure of the model's confidence in the predictions. An alternative approach to BNNs is Bayesian model averaging, in which predictions of multiple models are averaged. In practice, several methods of applying Bayesian inference to uncertainty estimation in deep learning have been proposed. Those include Markov chain Monte Carlo,^43^ variational inference,^44^ Monte Carlo dropout (MC dropout),^41^ and deep ensembles.^45^

Predictive uncertainty can be decomposed into two components: aleatoric, which captures inherent randomness in the data (such as noise in the labels), and epistemic, which represents the uncertainty of the model (e.g., when making predictions on out-of-distribution samples).^46^ Kendall et al.^42^ proposed an uncertainty decomposition method using a neural network that outputs two values representing the mean and variance of the prediction and using MC dropout to approximate Bayesian inference. A similar approach was used by Kwon et al.,^47^ who proposed a new method for estimating aleatoric and epistemic uncertainties using MC dropout. Fort et al.^48^ recently demonstrated that deep learning ensembles with random initializations allow for a more robust approach to uncertainty estimation. This is attributed to the fact that deep ensembles explore entirely different modes, whereas methods which are based on exploring the subspace of a single model (such as MC dropout) converge to a single mode.

Following Lakshminarayanan et al.,^45^ we used deep ensembles as an alternative to Bayesian inference. In our experiments, all models were trained for the same duration of 30 epochs. We trained separate ensembles of M = 10 models for each of the studied cohorts and an additional one for the combined cohort (CALM + PREV + UK-AIH). Overall, 12 ensembles of M = 10 models each were trained. Slides from the HepaT1ca cohort were retained as a separate validation set.

Given an ensemble Ω of M trained models $\Omega ={\left\{\omega_{m}\right\}}_{m=1}^{M}$, we computed the pixel-wise collagen likelihood as the average $\hat{p}$ of the ensemble predictions $p_{m}$ for an input pixel i.

$$\begin{array}{r} p_{m}=p\left(y|i,\omega_{m}\right)\\ \hat{p}=\frac{1}{M}\sum_{m=1}^{M}p_{m}. \end{array}$$

We then used the method proposed by Kwon et al.^47^ to estimate the pixel-wise predictive uncertainty for an input pixel i, and the output prediction y as the variance of y given i over the predictive distribution $p\left(y|i,\Omega \right)$:

$$\begin{array}{r} {\mathrm{Var}}_{p\left(y|i,\Omega \right)}\left(y\right)=\underset{\text{aleatoric}}{\underset{\underbrace{}}{\frac{1}{M}\sum_{m=1}^{M}\left[p_{m}\left(1-p_{m}\right)\right]}}+\underset{\text{epistemic}}{\underset{\underbrace{}}{\frac{1}{M}\sum_{m=1}^{M}{\left(p_{m}-\hat{p}\right)}^{2}}}. \end{array}$$

Two approaches for computing collagen percentage area in a tile were employed; the first, made use of the mean ensemble prediction P, and the other of the voted ensemble segmentation B. The mean collagen prediction P for image I with dimensions W,H is given by the equation:

$$\begin{array}{r} P=\frac{1}{\mathrm{WH}}\sum_{x=1}^{W}\sum_{y=1}^{H}\hat{p}\left(x,y\right) \end{array}.$$

Collagen segmentation value $\hat{b}$ was obtained for each pixel via thresholding of the corresponding ensemble prediction at collagen likelihood equal or higher than 0.5. Let $\hat{p}\left(x,y\right)$ be the ensemble prediction value for pixel i at location $\left(x,y\right)$ in the image I, and let $\hat{b}\left(x,y\right)$ be the corresponding binary value after thresholding. Hence, $\hat{b}\left(x,y\right)$ can be defined as:

$$\begin{array}{r} \hat{b}\left(x,y\right)=\left\{\begin{array}{ll} 1, & \text{if}\;\hat{p}\left(x,y\right)\geq 0.5\\ 0, & \text{otherwise}. \end{array}\right) \end{array}$$

The segmented collagen area proportion B is given by:

$$\begin{array}{r} B=\frac{1}{\mathrm{WH}}\sum_{x=1}^{W}\sum_{y=1}^{H}\hat{b}\left(x,y\right). \end{array}$$

The uncertainty in the mean tile collagen prediction is reported as the interquartile range (IQR) of the ensemble collagen predictions P. Similarly, the uncertainty of the segmented collagen area in a tile is reported as the IQR of the collagen segmentation maps produced by thresholding of the predictions by each of the models in the ensemble.

## Results

The validation metrics for the trained segmentation models are summarized in Table 5. We provide the scores separately for ensembles trained and validated on the individual study cohorts and for the ensemble models trained on the pooled cohort (CALM + PREV + UK-AIH).

**Table 5:** Segmentation network Dice scores on validation tiles (M = 10 ensemble average ± standard deviation).

Training set	CALM	PREV	UK-AIH	HepaT1ca
*Individual cohorts*				
U-Net Tiny	0.847 ± 0.022	0.877 ± 0.012	0.793 ± 0.036	–
U-Net Mini	**0.905** ± **0.026**	**0.882** ± **0.005**	**0.852** ± **0.019**	–
Att U-Net	0.820 ± 0.022	0.825 ± 0.059	0.759 ± 0.070	–
*Pooled cohort*				
U-Net Tiny	0.823 ± 0.021	0.861 ± 0.013	0.767 ± 0.037	**0.594** ± **0.075**
U-Net Mini	**0.834** ± **0.029**	**0.864** ± **0.014**	**0.795** ± **0.035**	0.555 ± 0.104
Att U-Net	0.785 ± 0.036	0.834 ± 0.017	0.623 ± 0.067	0.539 ± 0.123

The U-Net Mini has achieved the highest validation metric scores in all the assigned tasks. It can be seen that for all model architectures, there is a drop in Dice scores between the ensembles trained on individual cohorts and those trained on the pooled cohort. The higher Dice scores for individual cohorts can be explained by the models overfitting to the data within each of the cohorts. It can be seen that whereas very high Dice scores are achieved for the CALM and PREV cohorts, performance of each of the network architectures is slightly worse for the UK-AIH cohort. This can be explained by the fact that the UK-AIH samples were collected retrospectively from a large number of hospitals and are characterized by frequent staining artifacts and large intracohort differences in slide color as shown in Fig. 1 and quantitatively in Fig. 6, Fig. 7. As expected, the performance of the segmentation models is significantly lower for the unseen cohort (HepaT1ca). This drop in Dice scores is likely driven by cohort-specific differences in staining, color profiles, and imaging protocols not represented in the training data. Whereas our ensemble models were trained to capture variability across multiple centers, HepaT1ca remains an external dataset with distinct characteristics. This performance drop highlights the limits of generalizability even in diverse training settings and motivates future exploration of robustness-enhancing techniques.

**Fig. 9:**
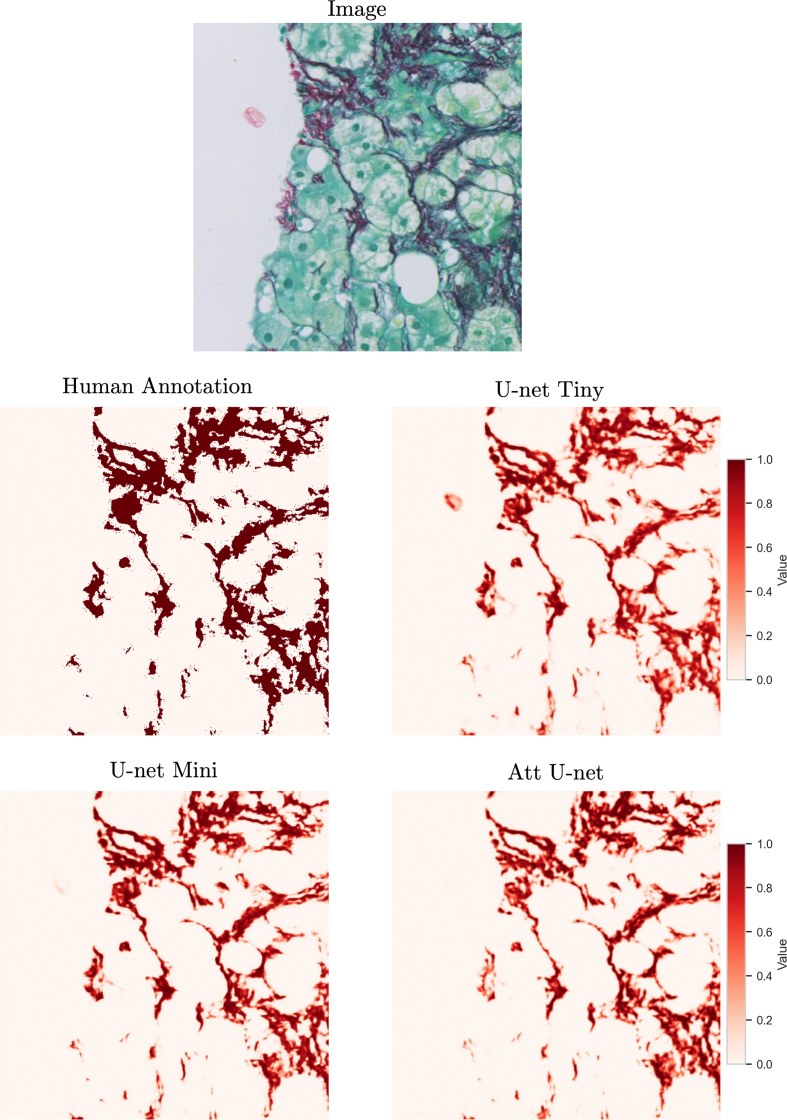
Collagen prediction maps output by the tested segmentation U-Net models. The human annotation used as ground truth for model validation contains noisy false-positive pixels around the annotated fibers. Each prediction map shows an output prediction from a single model trained on the pooled cohort. It is difficult to see any differences in the quality of the collagen maps predicted by different model types.

Fig. 9 shows model predictions for an example tile from the CALM cohort. It can be seen that the human annotation used as ground truth for model validation contains noisy false-positive pixels around the annotated fibers. These noisy labels can be attributed to falsely positive labeling by the annotator (MW) of the JPEG compression-induced noise in the original WSI. All prediction maps generated by the tested U-Net models provide a realistic representation of the collagen pattern in the original image. It can be seen that neither of the trained segmentation models has replicated the image compression noise in the final prediction, which is a desirable outcome. It is difficult to notice any visual differences in the quality of the prediction maps output by each of the models. This shows that each of the tested networks is appropriate for the task of collagen segmentation.

### Uncertainty computation

**Fig. 10:**
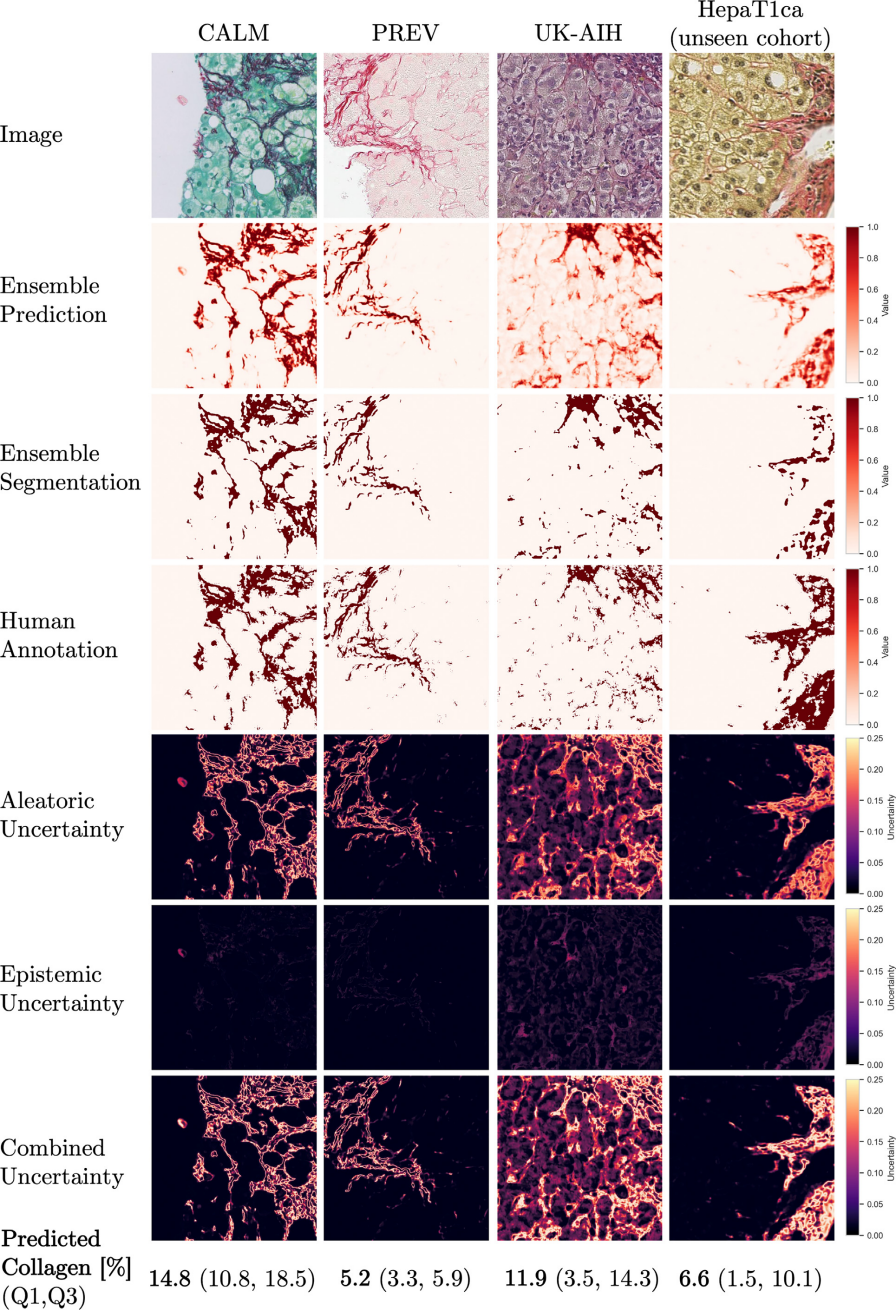
The results of U-Net Mini ensemble model prediction with uncertainty estimations for examples of unseen tiles from each of the cohorts. Tiles with low contrast of stained collagen and unseen colors are characterized by high ensemble model uncertainty.

**Fig. 11:**
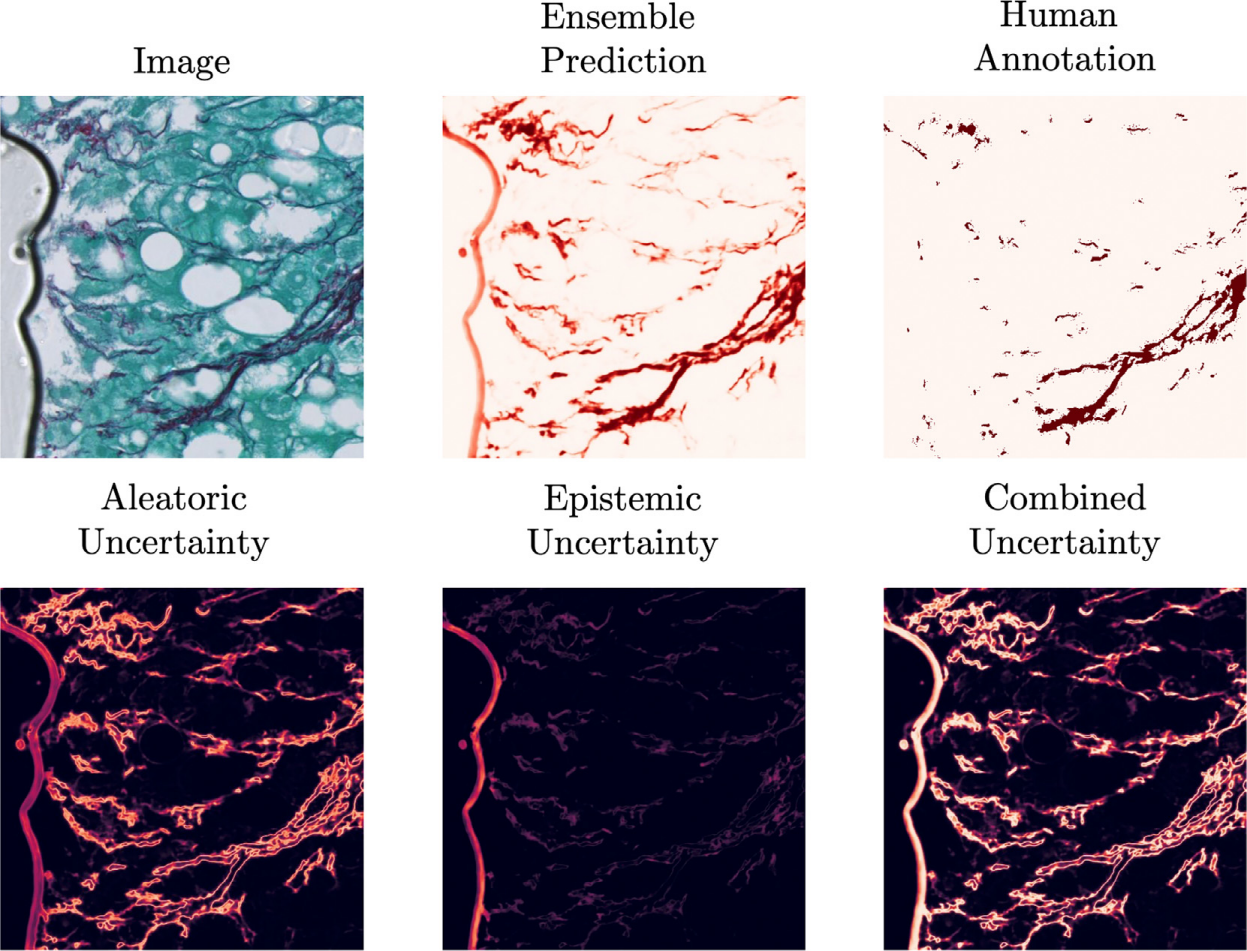
An example image prediction tile with high epistemic uncertainty. The air bubble visible in the original tile is highlighted in the epistemic uncertainty map, which visualizes out of distribution data.

Our ensemble-based uncertainty estimates follow the framework introduced by Lakshminarayanan et al.,^45^ which demonstrated that deep ensembles provide a practical and well-calibrated alternative to Bayesian inference for predictive uncertainty estimation. In this context, the ensemble variance visualized in Fig. 10, Fig. 11 offers a qualitative view of calibration behavior analogous to reliability plots used in classification tasks.

Fig. 10 shows the results of model predictions and uncertainty maps for the U-Net Mini ensemble trained on the pooled cohort. It can be seen that almost all the aleatoric uncertainty is concentrated on the boundaries of collagen fibers. This is expected, as boundary pixels are commonly affected by partial volume effects. Because of this phenomenon, the aleatoric uncertainty is directly tied to the length of the collagen outline in the tile. We observe that tiles with little to no collagen are characterized by uncertainty values near to 0 and tiles with much collagen have high inherent collagen area uncertainty. Uncertainty is computed for all inputs, including slides of suboptimal quality, as part of assessing model robustness under realistic multi-center conditions. Rather than serving as a rejection criterion, the uncertainty maps indicate which regions within a slide may yield unreliable measurements, complementing rather than replacing conventional quality control.

Table 6 presents the mean ensemble uncertainty scores obtained from model predictions on the validation set. It can be seen that the values of epistemic uncertainty are very low compared to the values of aleatoric uncertainty for all the image ensembles. We conclude that the uncertainty of the collagen area measurement primarily depends on the characteristics of a given image. The variability of the measured collagen percentage due to differences in predictions by individual segmentation models within each ensemble is relatively very small.

**Table 6:** Ensemble uncertainty scores on the validation set for each of the trained model types (M = 10 ensemble average ± standard deviation).

Model ensemble	U aleatoric	U epistemic	U combined
U-Net Tiny	0.020 ± 0.025	0.002 ± 0.003	0.022 ± 0.027
U-Net Mini	0.017 ± 0.022	0.002 ± 0.003	0.019 ± 0.025
Att U-Net	0.024 ± 0.036	0.003 ± 0.006	0.027 ± 0.041

Fig. 11 shows ensemble model prediction and uncertainties for a tile with a common imaging artifact, an air bubble. The bubble can be clearly seen in the epistemic uncertainty map, meaning that some of the models in the ensemble have classified its pixels as regions of collagen, and some did not. The visualization of epistemic uncertainty can provide the pathologist with a map of regions in the slide, which were not represented in the model training set, and therefore are likely to contain prediction errors. These examples illustrate the practical value of uncertainty quantification: regions with high epistemic uncertainty frequently correspond to visible artifacts or staining inconsistencies, indicating a higher likelihood of segmentation errors. Whereas a quantitative correlation between uncertainty magnitude and segmentation accuracy would require ground-truth fibrosis scoring across all cohorts, the qualitative consistency observed in Fig. 10, Fig. 11 demonstrates that uncertainty maps effectively highlight potentially unreliable regions. In this way, uncertainty estimation not only improves interpretability but also provides a mechanism to flag regions requiring closer manual inspection.

### Interpreting uncertainty

The results of uncertainty scores computed for each of the cohorts are provided in Table 7.

**Table 7:** Ensemble prediction uncertainty scores for validation samples from each of the cohorts, U-Net Mini (M = 10 ensemble average ± standard deviation).

Training set	CALM	PREV	UK-AIH	HepaT1ca
*Individual cohorts*				
U aleatoric	0.016 ± 0.016	0.006 0.008	0.024 0.020	–
U epistemic	0.002 ± 0.003	0.001 0.003	0.003 0.005	–
*Pooled cohort*				
U aleatoric	0.026 ± 0.028	0.008 ± 0.011	0.044 ± 0.033	0.018 ± 0.022
U epistemic	0.002 ± 0.002	0.001 ± 0.001	0.001 ± 0.005	0.002 ± 0.004

**Fig. A.2:**
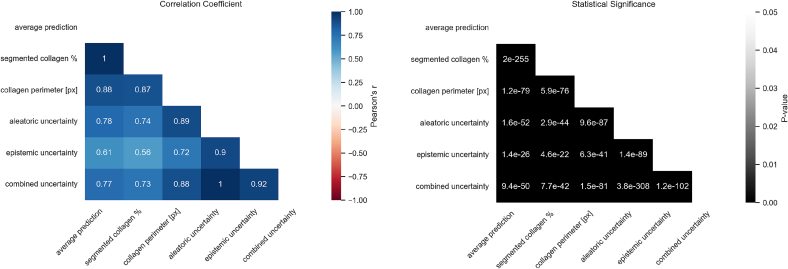
Correlation coefficients and corresponding statistical significance values for the prediction parameters of the model trained on the pooled cohort. The values are reported for predictions obtained using the U-Net Mini ensemble.

The PREV cohort has the lowest aleatoric uncertainty values of the cohorts studied. The aleatoric uncertainty is highly correlated to the area of collagen in an image (see Fig. A.2) and therefore, the lower uncertainty in the PREV cohort can be partly explained by the lower content of collagen in this cohort overall (see Table 3).

Interestingly, after cohort pooling the mean aleatoric uncertainty scores increase for all the models, especially in the cohorts where more than one stain color was used (i.e., CALM and UK-AIH). This indicates that the segmentation models introduced to different variants of collagen staining have lower confidence at the collagen boundaries, where more than one stain is present. Such interpretation would correspond to the intuition that placing additional stains on the tissue specimen introduces additional sources of variability in the data. The highest aleatoric uncertainty values are reported for the UK-AIH cohort in which the purple hematoxylin staining was used in the PSR slides. Collagen tiles in this cohort have overall a lower visual contrast than those from other cohorts.

It can be seen that the correlation between the average prediction P and the segmented collagen B is equal to 1, meaning that these measures can be considered equivalent to each other (Fig. A.2). This means that it may be acceptable to present pathologists with the collagen prediction maps rather than the binarized segmentation maps. The prediction maps have a closer resemblance to the original input image as they capture the color intensity gradients characteristic of collagen fibers of different thicknesses. Collagen prediction maps are also visually more similar to the images acquired using SHG/TPEF microscopy, where similarly, an inferred collagen density map rather than a binary segmentation map is obtained.^49^ Such continuous collagen prediction maps could be interpreted as a kind of “ideal” stain deconvolution, where only pixels containing any collagen can have positive values, and regions of tissue which have no collagen (i.e., cell cytoplasm, cell nuclei) have a collagen probability of 0.

From a practical standpoint, the acceptable level of uncertainty will depend on the intended use of the segmentation output. Based on the ensemble statistics in Table 7, epistemic uncertainty values for the U-Net Mini trained on the pooled cohort were typically around 0.002. Predictions with markedly higher epistemic uncertainty may indicate out-of-distribution image regions or unreliable model behavior and should be prioritized for review. Aleatoric uncertainty, by contrast, reflects intrinsic image ambiguity (e.g., collagen fiber boundaries) and is therefore less suitable for defining reliability thresholds. In practice, uncertainty maps can serve as an intuitive visual cue to guide users toward regions that may warrant closer inspection.

## Discussion

The problem of performing quantitative histological analysis on differently stained slides is well recognized. Early stain normalization relied on color deconvolution,^6^^,^^7^^,^^50^ but its assumptions of white-light illumination and stable stain vectors often fail in WSI.^9^ Later, color-transfer and generative approaches, including histogram matching,^10^ sparse autoencoders,^11^ and CycleGAN-based models,^12^, ^13^, ^14^, ^15^, ^16^ achieved visually realistic transformations, yet their biological fidelity remains uncertain.

Whereas domain adaptation strategies, including stain normalization and color transfer, have been proposed to improve cross-site generalizability, their impact on prediction and segmentation accuracy remains limited.^51^^,^^52^ In practice, deep ensembles trained on diverse data may offer greater robustness, particularly when supported by uncertainty quantification. Nonetheless, exploring domain adaptation remains a potential direction for future work in settings with limited training diversity or highly divergent staining.

Automated quality-control tools such as HistoQC are highly effective for detecting slide-level artifacts (e.g., tissue folds, pen marks, and out-of-focus regions) and can also report basic color and intensity statistics.^17^ However, these tools are primarily designed for identifying outliers or artifacts rather than systematically quantifying intercohort staining variability. Our color-space analysis, therefore, provides a complementary perspective, enabling cohort-level assessment of staining and acquisition differences. Future studies may benefit from combining color-space characterization with automated QC metrics to provide both global and local assessments of data quality.

Beyond quality control, uncertainty estimation offers an orthogonal way to assess the reliability of automated predictions. Uncertainty estimates can inform data-curation workflows by directing human annotation toward the most ambiguous image regions, supporting active-learning paradigms that improve efficiency without compromising quality. In this context, uncertainty estimation should not be viewed as a replacement for quality control or data curation, but as a complementary mechanism for assessing the trustworthiness of model predictions on heterogeneous, real-world data. By quantifying prediction confidence directly from the model output, uncertainty maps provide a means to contextualize results even when input quality varies across centers.

Whereas the U-Net architecture is long established in biomedical image segmentation, it remains a robust reference model valued for its stability, interpretability, and extensive validation across histopathology tasks. Our aim in this study was not to benchmark architectures, but to evaluate the estimation and interpretation of predictive uncertainty. Using the U-Net provided a controlled and transparent framework for ensemble-based uncertainty analysis across diverse staining conditions. We acknowledge that newer architectures, including transformer-based and foundation-model approaches, may further improve cross-site generalization.^53^^,^^54^ However, our results show that even this lightweight and classical model achieves strong multi-center performance (Dice 0.83–0.90) with modest computational cost, suggesting that uncertainty estimation itself—rather than architectural novelty—offers substantial benefits for model interpretability and reliability.

From a computational standpoint, ensemble-based uncertainty estimation inevitably increases training and inference time compared with a single model. However, the overhead is moderate: inference of a 512 × 512 px tile required approximately 2.5 s on a Quadro RTX 6000 GPU (8). Given the interpretability and calibration advantages of ensemble methods, this cost is acceptable for research and batch-processing workflows, particularly in settings where throughput is less critical than reliability.

The extreme staining variability observed across cohorts—particularly in the number and type of counterstains—highlights the challenge of developing a unified segmentation approach under real-world conditions. Rather than excluding these divergent slides, we deliberately retained them to assess how ensemble-based uncertainty estimation performs across heterogeneous inputs. Our findings indicate that, despite substantial color variation, the model maintained acceptable cross-cohort performance, and uncertainty maps successfully identified regions where predictions were less reliable. This suggests that uncertainty estimation can complement stain harmonization efforts by providing a mechanism to interpret and manage variability, whereas future work should aim to integrate these strategies through domain adaptation or foundation-model frameworks.

Although non-invasive imaging methods continue to advance,^55^ histology remains essential for definitive fibrosis assessment. By improving the reliability and interpretability of slide-based quantification, our approach supports this diagnostic foundation and facilitates comparison with emerging non-invasive metrics.

## Conclusions

This study demonstrates that ensemble-based uncertainty estimation strengthens quality control and supports more reliable deployment of automated collagen quantification in multi-center liver biopsy cohorts. Across four independent PSR-stained cohorts, we observed pronounced staining heterogeneity. Within this setting, the ensemble U-Net achieved strong segmentation performance in the training cohorts (Dice 0.83–0.90) and generated informative uncertainty maps. These maps captured both image-intrinsic ambiguity and model-related uncertainty, allowing direct visualization of prediction confidence at the pixel level. Segmentation performance decreased in the unseen HepaT1ca cohort, reflecting substantial staining divergence. The uncertainty maps nevertheless remained spatially informative and preserved interpretability of model behavior under domain shift.

The combined use of ensemble prediction, uncertainty mapping, and intercohort color-distribution analysis enabled transparent evaluation of model behavior across centers. Explicit quantification of prediction confidence extends validation beyond aggregate performance metrics and supports informed interpretation of automated CPA measurements. In practice, epistemic uncertainty provides a mechanism for identifying unreliable predictions and prioritizing them for expert review, reinforcing existing quality-control procedures.

Future multi-center studies will benefit from incorporating structured characterization of stain differences and systematic analysis of uncertainty-associated failure modes alongside conventional performance metrics. Integration of such evaluation strategies with continued stain harmonization efforts and foundation-model representations trained on diverse histopathology data will further strengthen cross-center reliability. Collectively, these approaches advance the development of more transparent, trustworthy, and reproducible computational pathology workflows.

## Declaration of use of AI

During the preparation of this work, the authors used ChatGPT (OpenAI, San Francisco, USA) in order to assist with language editing and improving clarity. After using this tool, the authors reviewed and edited the content as needed and take full responsibility for the content of the published article.

## Declaration of competing interest

The authors declare that they have no known competing financial interests or personal relationships that could have appeared to influence the work reported in this article.

## Figures and Tables

**Table A.1:** Training and inference time comparison for the trained models (M = 10 ensemble average ± standard deviation).

Model	Training [s]	Inference [s]
U-Net Tiny	417.7 ± 16.0	1.3 ± 0.2
U-Net Mini	1358.8 ± 24.5	2.5 ± 0.6
Att U-Net	1590.4 ± 37.0	3.3 ± 0.8

**Fig. A.1:**
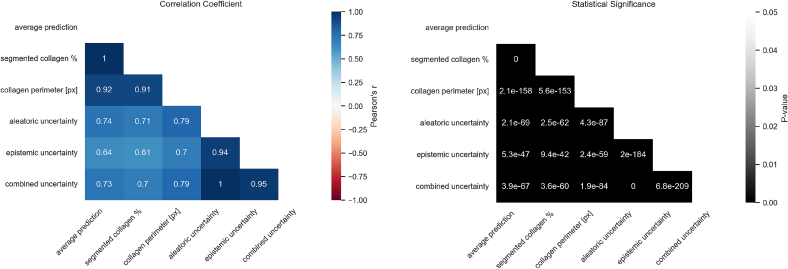
Correlation coefficients and corresponding statistical significance values for the prediction parameters of the model trained on the individual cohorts. The values are reported for predictions obtained using the U-Net Mini ensemble.

## Data Availability

The source code for the slide color analysis tool is available under appendix.

## Appendix A Appendix

### A.1 Processing time

Table A.1 shows a comparison of the training and inference times for each of the tested models. The times are reported for the processing of the training set (training over 30 epochs) and of the validation set, respectively. It can be seen that tile processing time is strongly related to the size of the network used, with U-Net Tiny outperforming other models in our study. Processing time is an important factor to consider when choosing deep learning models for use in clinical practice.

### A.2 Correlation statistics

Fig. A.1, Fig. A.2 show the correlation statistics for computed collagen areas (the average prediction P and the segmented collagen area B), as well as uncertainties for the models trained on the individual cohorts and the model trained on the pooled cohort, respectively.

## Footnotes

h https://github.com/stefano-malacrino/wsi-reader.

i https://github.com/cgohlke/tifffile.

j https://github.com/yingkaisha/keras-unet-collection.
